# Influence of 4-week multi-strain probiotic administration on resting-state functional connectivity in healthy volunteers

**DOI:** 10.1007/s00394-018-1732-z

**Published:** 2018-05-30

**Authors:** Deepika Bagga, Christoph Stefan Aigner, Johanna Louise Reichert, Cinzia Cecchetto, Florian Ph. S. Fischmeister, Peter Holzer, Christine Moissl-Eichinger, Veronika Schöpf

**Affiliations:** 1 Institute of Psychology, University of Graz, Universitätsplatz 2, 8010 Graz, Austria; 2 BioTechMed, Graz, Austria; 3 Institute of Medical Engineering, Graz University of Technology, Graz, Austria; 4 Otto Loewi Research Centre, Pharmacology Section, Medical University of Graz, Graz, Austria; 5 Department of Internal Medicine, Medical University of Graz, Graz, Austria

**Keywords:** Probiotics, Resting-state, Diffusion, Gut–brain axis, MRI, Salience

## Abstract

**Purpose:**

Experimental investigations in rodents have contributed significantly to our current understanding of the potential importance of the gut microbiome and brain interactions for neurotransmitter expression, neurodevelopment, and behaviour. However, clinical evidence to support such interactions is still scarce. The present study used a double-blind, randomized, pre- and post-intervention assessment design to investigate the effects of a 4-week multi-strain probiotic administration on whole-brain functional and structural connectivity in healthy volunteers.

**Methods:**

Forty-five healthy volunteers were recruited for this study and were divided equally into three groups (PRP: probiotic, PLP: placebo, and CON: control). All the participants underwent resting-state functional MRI and diffusion MRI brain scans twice during the course of study, at the beginning (time point 1) and after 4 weeks (time point 2). MRI data were acquired using a 3T whole-body MR system (Magnetom Skyra, Siemens, Germany).

**Results:**

Functional connectivity (FC) changes were observed in the default mode network (DMN), salience network (SN), and middle and superior frontal gyrus network (MFGN) in the PRP group as compared to the PLP and CON groups. PRP group showed a significant decrease in FC in MFGN (in frontal pole and frontal medial cortex) and in DMN (in frontal lobe) as compared to CON and PLP groups, respectively. Further, significant increase in FC in SN (in cingulate gyrus and precuneus cortex) was also observed in PRP group as compared to CON group. The significance threshold was set to *p* < 0.05 FWE corrected. No significant structural differences were observed between the three groups.

**Conclusions:**

This work provides new insights into the role of a multi-strain probiotic administration in modulating the behaviour, which is reflected as changes in the FC in healthy volunteers. This study motivates future investigations into the role of probiotics in context of major depression and stress disorders.

## Introduction

The characterization of gut microbiome a decade ago has added a long-overlooked aspect to the complex bidirectional signalling between brain and gut [1]. This interaction, known as ‘gut–brain axis’ has been shown to link the cognitive and emotional centres of brain with the intestinal functions [2]. The gut microbiota plays a prominent role in these interactions by regulating behaviour and brain processes, that is, stress responsivity [3], anxiety-related behaviours [4], pain perception [5], and social cognition [6], as shown by intriguing experimental investigations in rodents. In addition to the emotional processing, gut microbiota have also been shown to play an important role in modulating brain biochemistry and brain plasticity. For example, Hoban and colleagues showed that the gut microbiota regulates the expression of genes linked to myelination and myelin plasticity in prefrontal cortex [7]. On a similar note, a role of gut microbiota in altering the central GABA (gamma amino butyric acid) receptor expression has also been demonstrated [8]. Although most of the evidence for an influence of gut microbiota on brain and behaviour is based on our understanding of rodent studies, initial studies in humans seem to support the notion that there exists a similar relationship between our gut microbes and brain and behaviour. For instance, consumption of *Lactobacillus* and *Bifidobacterium* strains by healthy volunteers was found to influence the scores of stress and anxiety-related questionnaires [9–11]. However, as the assessment was based on self-reported measures in all these studies, caution is warranted when drawing firm conclusions. Furthermore, some recent studies have employed neuroimaging techniques such as task-based functional MRI and resting-state fMRI to better understand the physiological pathways involved in gut–brain communications and their influence on brain function [12, 13]. A task-based fMRI study conducted in our group [12] demonstrated that a 4-week multi-strain probiotic administration influences brain activation patterns associated with emotional decision-making and recognition memory tasks in healthy volunteers. Another fMRI study by Tillisch and colleagues showed that the ingestion of *Lactobacillus* and *Bifidobacterium* species for 4 weeks by healthy women altered the brain activity in insula, somatosensory cortex and periaqueductal gray brain regions in response to an emotional attention fMRI task [13]. This study also investigated the corresponding functional connectivity (FC) changes in these regions using region of interest (ROI) analysis and reported changes in FC in midbrain regions. However, the influence of probiotic administration on whole-brain functional connectivity remains unclear. Furthermore, even when there is a considerable volume of preclinical literature indicating an influence of gut microbiome on brain structure, our understanding in human subjects in this context is limited to the observations in patients with irritable bowel syndrome (IBS) [14] and this is far from complete.

Numerous neuroimaging studies have revealed a strong relationship between structural integrity and functional connectivity (see review by Damoiseaux and Greicius [15]). Functional connectivity is most commonly calculated from resting-state fMRI and examines the similarities between spontaneous fluctuations that occur over time in distal grey matter regions [16] and diffusion MRI measures the structural integrity [17]. Considering the existing literature on the influence of probiotic administration on functional connectivity [13], a further investigation of the structural basis for these functional interactions would add valuable insights to our current understanding of the gut–brain interaction mechanisms.

In this study, we aimed at investigating the influence of a 4-week multi-strain probiotic administration on whole-brain functional connectivity in healthy volunteers. We hypothesized that the manipulation of gut microbiota by multi-strain probiotic ingestion will influence functional connectivity in the resting-state networks (RSNs) mediating emotional and higher order cognitive functions. We suspect that salience network, executive network and default mode network are of particular interest, considering their role in mediating these processes. Furthermore, we also hypothesized that the probiotic intervention will influence the underlying white matter architecture associated with the functional connectivity networks. To test these hypotheses, we performed resting-state fMRI and diffusion MRI scanning at two time points: at baseline (time point 1) and after 4 weeks (time point 2).

## Methods

### Subjects and study design

The present study used a double-blind, randomized, pre- and post-administration (4 weeks) assessment design. Forty-five right-handed healthy participants (mean age (years) = 26.24, SD = 4.76; 23 female; age group 20–40 years) were recruited for this study via university email lists, flyers, and word of mouth. The participants were divided equally into three groups: probiotics (PRP) group (which took the probiotics product), placebo (PLP) group (which took the placebo product), and control (CON) group (with no product). This study was conducted in accordance with the principles of the Declaration of Helsinki and written informed consent was obtained from all participants prior to participation. The local ethics committee of the University of Graz, Austria, approved the study. Exclusion criteria were MR incompatibility, substance abuse, use of antibiotics or probiotics (in the last 3 months), and CNS trauma/disorders.

All the participants underwent MRI scanning at baseline (time point 1) and after 4 weeks (time point 2). The appointments for the second scanning were planned well in advance, to assure equal intervals between the first and second scanning session for all the participants. During this period, all participants were instructed to fill in a daily diary about their gastrointestinal symptoms and details of probiotic/placebo intake [time of intake, method of intake (with milk/water/juice)]. Further, participants were instructed to maintain their usual diet and lifestyle habits during the 4-week period. Any deviation from this was instructed to be recorded in the daily diary for later assessment. Additional questionnaires were incorporated into a daily diary and participants were briefed about the instructions to fill these out at the beginning of the study.

This study is part of another research project which investigated changes in behaviour (using self-reported questionnaires) and brain function (using task-based fMRI) following probiotic intake. Therefore, details of participant characteristics and assessments if not necessary for understanding are reported elsewhere [12].

### Study product and administration

The probiotic formulation used for this study was Ecologic®825 (manufactured by Winclove Probiotics, The Netherlands, and available on the market as OmniBiotic^®^ Stress Repair, Institut Allergosan, Austria). Daily doses were supplied as sachets, each containing 3 g freeze-dried powder. The product (7.5 × 10^6^ CFU/g) is composed of nine bacterial strains, namely *Lactobacillus casei* W56, *Lactobacillus acidophilus* W22, *Lactobacillus paracasei* W20, *Bifidobacterium lactis* W51, *Lactobacillus salivarius* W24, *Lactococcus lactis* W19, *Bifidobacterium lactis* W52, *Lactobacillus plantarum* W62 and *Bifidobacterium bifidum* W23. The placebo formulation was also supplied as sachets of 3 g freeze-dried powder composed of the carrier of probiotic product: maize starch and maltodextrins. The placebo was matched for colour, texture, and smell to the probiotic product, but contained no bacteria. At the time of first scanning, participants were provided with the product (probiotic or placebo) for the 4-week intervention. The participants were instructed to consume the product once a day (dissolving in milk or lukewarm water) preferably in the morning or before going to bed. No information was provided to the participants about the different types of intervention (probiotics vs. placebo) or the study hypothesis.

### MRI acquisition

All the participants were assessed twice: at the beginning (time point 1) and after 4 weeks (time point 2). The MRI data were acquired using a 3T whole-body MR system (Magnetom Skyra, Siemens, Germany) with a circularly polarized 32-channel matrix head coil and 45mT/m actively shielded gradient system. To minimize head movements, participants lay supine with their heads immobilized using foam pads. For anatomical reference, a high-resolution T1-weighted 3D gradient echo sequence (MPRAGE: Magnetization Prepared Rapid Acquisition Gradient Echo, 192 sagittal slices, field of view = 224 mm^2^, TE = 1.89 ms, TR = 1.68 s, slice thickness = 0.88 mm) image data set was acquired. Furthermore, diffusion-weighted data were acquired using echo-planar dual spin echo sequence in 64 directions. Diffusion-weighted acquisition parameters were: b-factor = 0 and 1000s/mm^2^, slice thickness = 2 mm, number of slices = 50, field of view = 240 mm^2^, TR = 6600 ms, and TE = 95 ms. Resting-state brain volumes were acquired using an echo planar T2*-weighted imaging sequence consisting of 32 interleaved slices (field of view = 256 mm^2^, TE = 27 ms, TR = 1.99 s, slice thickness = 4 mm, voxel size = 4*4*4 mm^3^). Scanning time for the resting-state sequence was 5 min and 24 s, during which the subjects were instructed not to think of anything in particular, not to move and not to fall asleep.

### Data analysis

#### Resting-state (RS) data

The RS data were pre-processed using the FMRI Expert Analysis Tool (FEAT), which is a part of FSL (FMRIB’s Software Library, http://www.fmrib.ox.ac.uk/fsl). For individual-level analysis, functional brain volumes were corrected for slice timing, smoothed with a Gaussian kernel of full-width at half-maximum of 5 mm [with high-pass temporal filtering (cut-off = 100 s)], registered to the individual’s structural scan (brain extracted using BET) (brain extraction tool [18]) and MNI (Montreal Neurological Institute) space using FMRIB’s Linear Image Registration Tool (FLIRT) [19]. While running FEAT, the Multivariate Exploratory Linear Optimized Decomposition into Independent Components (MELODIC) ICA (Independent Component Analysis) data exploration option was turned on (with ‘automatic dimensionality estimation’ option) to gain insight into unexpected artefacts or activation in the data. Further, the data sets derived from MELODIC were denoised using FIX (FMRIB’s ICA-based X-noiseifier) to further remove the noise components [20]. The denoised data were then decomposed into a set of 35 time courses and associated spatial maps (describing the temporal and spatial characteristics of underlying hidden signals) using MELODIC toolbox of FSL. Data were again denoised using FIX. Between-group data analysis was carried out using dual regression technique, which allows for voxel-wise comparisons of resting functional connectivity [21]. For this, MELODIC was run on the denoised data (all participants) in concat-ICA mode (multi-session temporal concatenation, no. of components = 20). For the randomize step delta (Δ) files were created by contrasting the time point 1 and time point 2 images of each participant. A general linear model was then defined to create multi-subject design matrix-defining groups (ΔPLP, ΔPRP, and ΔCON) and contrast files (ΔCON > ΔPLP, ΔCON < ΔPLP, ΔCON > ΔPRP, ΔCON < ΔPRP, ΔPLP > ΔPRP, ΔPLP < ΔPRP).

**Fig. 1:**
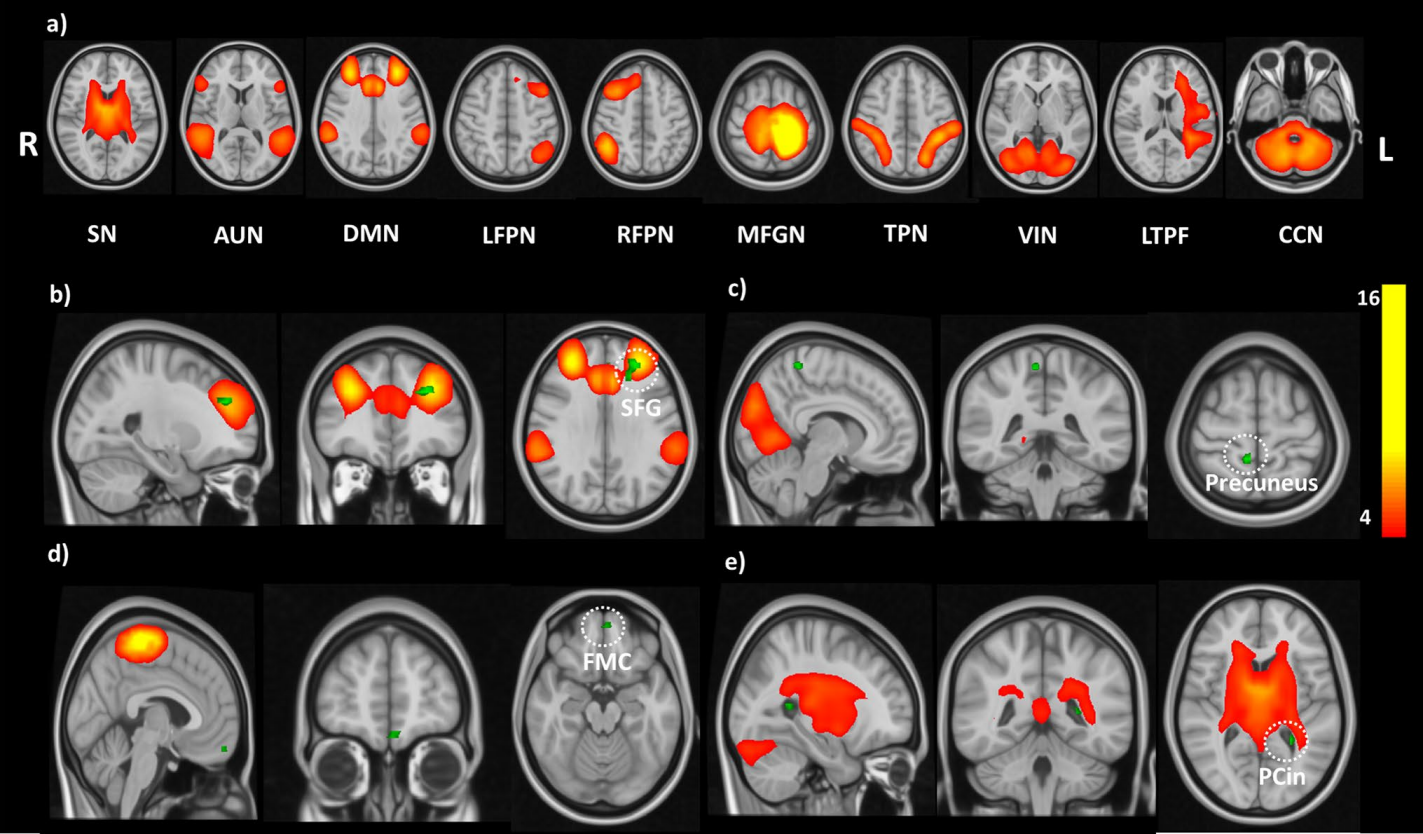
Resting-state results for the between-group (ΔCON, ΔPLP, ΔPRP) comparisons showing, (a) RSNs identified using ICA, which were used for the dual regression analysis; (b–e) randomized output for group comparisons thresholded at *p* < 0.05 FWE corrected; reduced FC in PRP group was observed in regions of (b) DMN, (c) VIN, (d) MFGN; increased FC in PRP group was observed in regions of (e) SN; results are shown on MNI 0.5 mm standard template. CON: no intervention; PLP: placebo; PRP: probiotic

The selection of spatial maps representing resting-state networks (Fig. 1a) was carried out by comparing to those found in the literature [22, 23]. Voxel-wise analyses of the group differences were carried out using FSL randomize non-parametric permutation testing with 10,000 permutations function per contrast [24]. Threshold-free cluster enhancement (TFCE) was used to control for multiple comparisons and the significance threshold was set to *p* < 0.05 FWE corrected. The Harvard–Oxford cortical atlas was used for anatomical labelling of ICA maps.

#### Diffusion-weighted data

Voxel-wise statistical analysis was performed using tract-based spatial statistics (TBSS) within FSL (http://www.fmrib.ox.ac.uk/fsl) [25]. The diffusion images were first corrected for susceptibility-induced and eddy current distortions. Non-brain tissue was removed from the images using BET implemented in FSL. A diffusion tensor model was fitted at each voxel of the corrected data using DTIFIT [26] allowing for the estimation of fractional anisotropy (FA) and mean diffusivity (MD). FA data of each participant were registered into a common space using nonlinear registration tool FNIRT using a b-spline representation of the registration warp field. Further, a mean FA image was created and thinned to create a mean FA skeleton, which represents the white matter tracts common to the whole group of participants. Each subject’s realigned FA maps were then projected onto these skeletons and subsequently fed into voxel-wise between-group statistics. Group differences in voxel-wise structural connectivity (FA and MD) were tested using a general linear model with 5000 permutations. Results were corrected for multiple comparisons, using FWE at *p* < 0.05 and TFCE.

## Results

### Probiotic intervention was associated with changes in the functional connectivity

Altogether ten independent components (ICs) were identified as resting-state networks (RSNs) from group MELODIC output. These components included salience network (SN), auditory network (AUN), default mode network (DMN), left fronto-parietal network (LFPN), right fronto-parietal network (RFPN), middle and superior frontal gyrus network (MFGN), task-positive network (TPN), visual network (VIN), left temporo-parietal–frontal network (LTPF) and cortico-cerebellar network (CCN) (Fig. 1a). These ICs were compared for differences in FC across the three groups: CON, PLP, and PRP. Significant changes in FC were observed in the default mode network (DMN), salience network (SN), visual network (VIN) and middle and superior frontal gyrus network (MFGN) when comparing the PRP group to the two other groups (PLP and CON). Specifically, PRP group showed a decreased FC in frontal pole and frontal medial cortex as compared to CON group within MFGN. Furthermore, as compared to PLP group, PRP group showed a decreased FC in VIN in brain regions, namely postcentral gyrus and precuneus and in DMN in frontal pole, SFG and paracingulate gyrus regions. We also observed an increase in FC in SN in PRP group as compared to CON group in brain regions, namely cingulate gyrus and precuneus cortex (see Fig. 1b–e; Table 1 for details).

**Table 1:** Summary of significant differences observed in resting-state networks across three groups

Contrasts	Network	Cluster voxels	MNI coordinates (*x, y, z*)			#Mean probability
ΔCON > ΔPRP	Middle and superior frontal gyrus network					
	Frontal pole	18	− 2	54	− 16	42.38
	Frontal medial cortex					20.88
ΔPRP > ΔCON	Salience network					
	Cingulate gyrus	11	− 26	− 50	12	18.1
	Precuneus cortex					7.20
ΔPLP > ΔPRP	Visual network					
	Postcentral gyrus	12	10	− 42	60	30.88
	Precuneus					23.66
ΔPLP > ΔPRP	Default mode network					
	Frontal pole	140	− 26	42	28	24.56
	Superior frontal gyrus					5.85
	Paracingulate gyrus					4.34

*CON* control, *PLP* placebo, *PRP* probiotic

*p* < 0.05 FWE corrected

### Probiotic intervention did not influence the structural connectivity

Analysis of regional differences in FA and MD using TBSS yielded no significant results after FWE correction for multiple comparisons. Even at a very lean threshold of *p* < 0.001 uncorrected, we only observed an insignificant increase in fractional anisotropy within the cingulum and the precuneus. However, this difference was only found when comparing the PRP group to the CON group.

## Discussion

The present study aimed at investigating the influence of a 4-week multi-strain probiotic administration in whole-brain functional and structural connectivity in healthy volunteers. Significant changes in FC were observed in PRP group as compared to PLP and CON groups, within the SN, DMN, VIN and MFGN resting-state networks. No corresponding structural differences were observed between the three groups. Our results reflect a change in FC in PRP group and are in line with the findings of Tillisch and colleagues [13], who demonstrated an influence of 4-week probiotic administration on FC associated with midbrain, insula and sensorimotor cortex brain regions. The present study was an attempt to further investigate this influence of probiotic administration on FC on whole-brain level.

In this study, the PRP group exhibited increased FC in the salience network (SN) as compared to the CON group in the cingulate gyrus and the precuneus cortex. A significantly decreased FC was observed in the DMN in PRP group as compared to PLP group in frontal pole, superior frontal gyrus (SFG) and paracingulate gyrus. Furthermore, we also observed a decreased FC in the MFGN in the PRP group as compared to the CON group. It is quite evident from the vast literature on resting-state fMRI studies that efficient behaviour involves the coordinated activity of large-scale networks and these interactions between the networks control and shape our behaviour. According to the triple network model proposed by Menon [27], the SN plays an important role in mediating the function of other networks and this is most evident when a rapid change in behaviour is required. SN dynamically controls the changes of FC between the DMN, which is related to the self-referential cognition, and central executive network (CEN), which is related to external-oriented tasks. Cingulate cortex is a key structure of SN and together with insula, it occupies an important position in initiating network switching between DMN and attentional networks [27, 28]. Changes in FC in this region in PRP group reflect an influence of probiotic administration on modulating behaviour and a shift towards efficient attentional control. These changes in FC in cingulate cortex were also reflected as changes in BOLD response in another emotional decision-making task-based fMRI study [12], reflecting the role of this region in emotional processes. In addition, coupled deactivation of DMN brain regions further reflects a shift towards efficient behavioural performance in PRP group. Studies have shown that the failure to deactivate the DMN is associated with attentional deficits [28]. Another significant observation in this study was a change in the FC in MFGN, which plays a key role in orienting of spatial attention [29], decision-making and cognitive control [30]. These results indicate an influence of probiotic administration not only on emotional processes, but going beyond extending into higher order cognitive processes. This fact is further supported by changes in FC in frontal lobe regions in PRP group as compared to PLP and CON group, as the frontal cortex is the key brain region associated with problem-solving, reasoning, attention, decision-making, learning, and creativity [31].

Next to functional changes, probiotics are expected to have structural changes as shown by preclinical studies [7]. However, even when lowering thresholds, we did not observe any structural connectivity differences associated with probiotic administration between the three groups. This suggests that a 4-week probiotic administration solely influences the behaviour. From our data, this change in behaviour is reflected as a modification of the interaction of resting-state networks and is not associated with structural changes. While we are well aware that the 4-week application period may be too short to induce any effect at structural level, a longer period of probiotic intake, for example, 8–12 weeks, was beyond the scope of this study.

Several plausible molecular mechanisms associated with gut–brain interactions have been discussed in the literature. Among these are the reciprocal connections of the vagus nerve as shown in preclinical studies [8], signalling molecules such as serotonin precursors, GABA and short-chain fatty acids [13], or via improving epithelial barrier function [32]. Based on these studies, one might expect these changes in functional connectivity in the present study to be mediated by chemicals, cytokines, hormones released by gut microbiota, which were manipulated with probiotic administration or via pathways mediated by vagus nerve.

Irrespective of the exact pathway, our results support the contention that the communication between gut microbiota and brain is a dynamic process, which can be modulated by a targeted intervention, which leads to changes in the behaviour and brain function. Although the efficacy of probiotics in the treatment of gastrointestinal diseases is well documented, preclinical studies have shown that probiotic intervention has the potential to modulate pain sensitivity, stress responsiveness, mood, anxiety and depressive symptoms in a beneficial manner. The results of the current study are relevant in guiding future clinical studies to address the question whether probiotic intervention might be of use as an alternative or adjunct strategy to treat depression and mood disorders. Neuroimaging techniques, specifically MRI, stand out as potential candidates for studying the effects of probiotic intervention in humans non-invasively using multimodalities, ranging from functional MRI, magnetic resonance spectroscopy to diffusion tensor imaging.

The present study and recent findings [12] have demonstrated that there is a close relationship between the effects of probiotic intervention on behavioural and neuroimaging readouts. However, studying the molecular mechanisms associated with probiotic intervention in humans is still an important question for future investigations in this field. Deeper understanding of these molecular mechanisms will definitely influence their clinical use in the future and potentially lead to new and specific formulations of probiotics, which might protect against a wide range of mood disorders and thus can revolutionize the field of therapeutics.
